# Neuronal Cav3 channelopathies: recent progress and perspectives

**DOI:** 10.1007/s00424-020-02429-7

**Published:** 2020-07-07

**Authors:** Philippe Lory, Sophie Nicole, Arnaud Monteil

**Affiliations:** 1 Institut de Génomique Fonctionnelle, CNRS, INSERM, University Montpellier, 141, rue de la Cardonille, 34094 Montpellier, France; 2 LabEx ‘Ion Channel Science and Therapeutics’ (ICST), Montpellier, France

**Keywords:** Calcium channels, T-type, Calcium channelopathies, Epilepsy, Ataxia, Autism, Schizophrenia, Primary aldosteronism

## Abstract

T-type, low-voltage activated, calcium channels, now designated Cav3 channels, are involved in a wide variety of physiological functions, especially in nervous systems. Their unique electrophysiological properties allow them to finely regulate neuronal excitability and to contribute to sensory processing, sleep, and hormone and neurotransmitter release. In the last two decades, genetic studies, including exploration of knock-out mouse models, have greatly contributed to elucidate the role of Cav3 channels in normal physiology, their regulation, and their implication in diseases. Mutations in genes encoding Cav3 channels (*CACNA1G*, *CACNA1H*, and *CACNA1I*) have been linked to a variety of neurodevelopmental, neurological, and psychiatric diseases designated here as neuronal Cav3 channelopathies. In this review, we describe and discuss the clinical findings and supporting in vitro and in vivo studies of the mutant channels, with a focus on de novo, gain-of-function missense mutations recently discovered in *CACNA1G* and *CACNA1H*. Overall, the studies of the Cav3 channelopathies help deciphering the pathogenic mechanisms of corresponding diseases and better delineate the properties and physiological roles Cav3 channels.

## Introduction

**Fig. 1:**
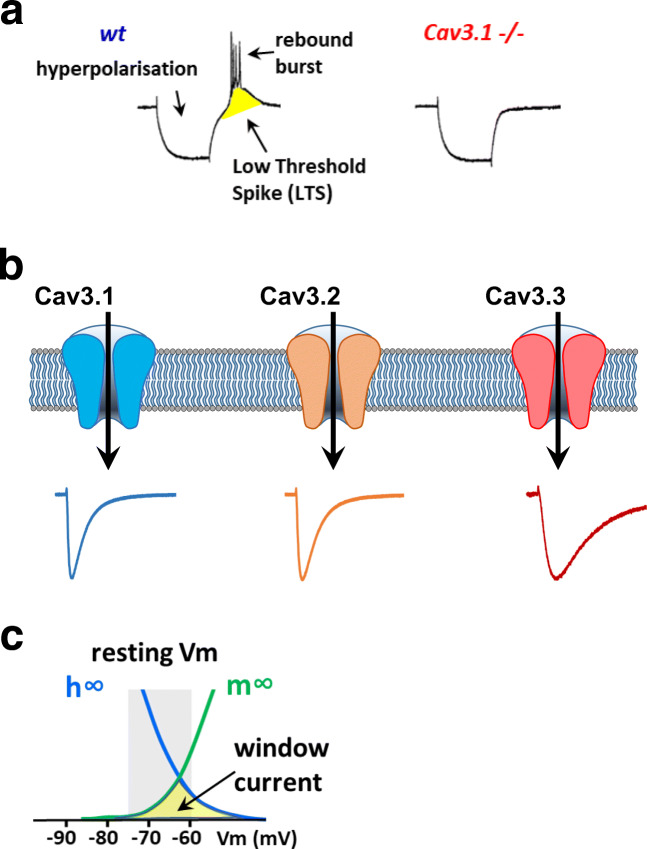
Electrophysiological properties of T-type/Ca3 channels. **a** Illustration of the implication of Cav3.1 channels in rebound burst firing in thalamocortical relay neurons, as reported in [81]. Hyperpolarization deinactivates T-type channels, which promotes low-threshold spike and rebound burst firing. This firing activity in completely lost in thalamocortical relay neurons from Cav3.1^−/−^ mice (for details, see [81]). **b** Current traces for Cav3.1, Cav3.2, and Cav3.3 channels obtained in HEK-293 cells, illustrating their differences in inactivation kinetics (see [13, 28]). **c** Illustration of the Cav3 window current that occurs in the range of the resting membrane potential

In the early 1980s, Llinas and Yarom [91] reported that hyperpolarization of inferior olivary neurons of the cerebellum could reveal a low-threshold Ca^2+^ conductance, which was inactivated at their resting membrane potential. The concept of low-voltage activated (LVA) Ca^2+^ current then arose in the neuroscience community as this conductance was described in many different types of neurons, including thalamic [90], sensory [19, 152], and hippocampal [165] neurons. This LVA Ca^2+^ current, also typical for its fast inactivation (*T*ransient) and small unitary conductance (*T*iny), was soon after named “T-type” [110, 112]. The unique voltage sensitivity of T-type Ca^2+^ channels is particularly well suited to regulate neuronal excitability and their oscillatory behavior near the resting membrane potential. A transient membrane hyperpolarization arising from inhibitory post-synaptic potentials (IPSPs) or activation of potassium (K^+^) channels deinactivates T-type channels. A subsequent rebound in the membrane potential triggers opening of T-type channels and favors a low-threshold spike (LTS) that initiates rebound burst firing (Fig. 1a). The role of T-type channels in bursting behavior is physiologically relevant, especially in sleep [5, 84] with the generation of sleep spindles. In the last two decades, following the molecular cloning of the Cav3 (T-type) channels in the 2000s, genetic studies have greatly contributed to elucidate the role of T-type channels in normal physiology, as well as to identify their implication in diseases. Notably, mutations in the genes encoding the Cav3 channels have been linked essentially to neurodevelopmental, neurological, and psychiatric diseases designated here as neuronal Cav3 channelopathies.

## Cav3 channels: from molecular to physiological diversity

### Cav3 molecular diversity

**Fig. 2:**
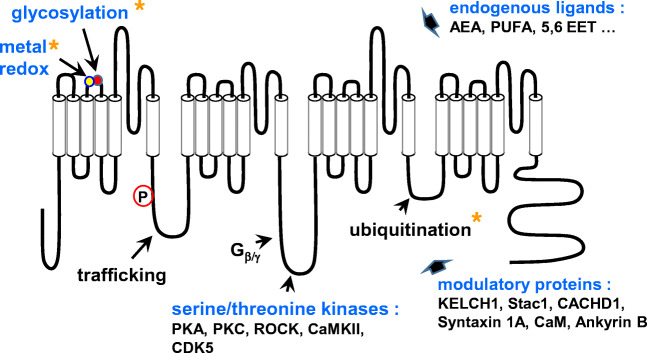
Schematic representation of the main Cav3 regulations (for previous reviews, see [29, 73, 75, 170]). The yellow asterisks point to the Cav3.2-selective regulations, including the metal/redox (His191, yellow circle) and glycosylation (Asn192, red circle) sites in S3–S4 extracellular linker of the domain I

**Fig. 3:**
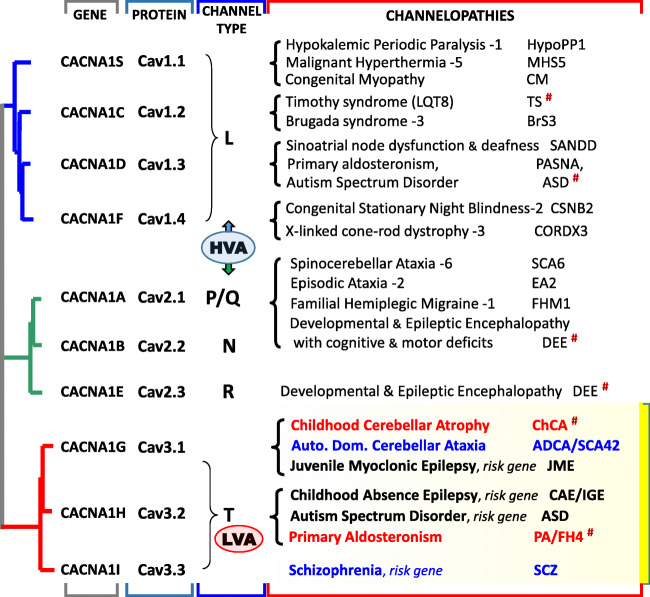
Cladogram representation of the Cav channel family including the gene names and the corresponding Cav subunits. HVA stands for high-voltage activated channels (L-, P/Q-, N-, and R-types) and LVA stands for low-voltage activated channels (T-type). The channelopathies column refers to the overall so-called Ca^2+^ channelopathies, with the detailed properties of the Cav3 channelopathies presented and discussed in the text. The diseases caused by mutations in the S6 segments of the corresponding Cav channels are indicated (#)

Before the cloning era, a diversity within T-type channels was already proposed, based on observed differences in inactivation properties and sensitivity to blockers, such as nickel (Ni^2+^) ions [74]. The first cDNA coding for the pore-forming subunit of a T-type channel was cloned in 1998 by Perez-Reyes and colleagues [119]. This was followed by extensive molecular cloning of several paralogs (isoforms) and orthologs, mainly in vetebrates, leading to the actual landscape of three genes (*CACNA1G*, *CACNA1H*, and *CACNA1I*) encoding the α1 subunit of T-type channels, Cav3.1 (α_1G_), Cav3.2 (α_1H_), and Cav3.3 (α_1I_), respectively (Figs. 1, 2, and 3) (for representative reviews, see [117, 118, 158]). The distinctive features of T-type channels making them well suited to regulate excitability (low voltage range for activation, ion selectivity, fast kinetics for activation and inactivation) are conserved in the most early-diverging animals, such as in *Trichoplax adhaerens*, which expresses a single Cav3 channel [135]. Hence, genome survey in *Salpingoeca rosetta* indicates that Cav3 channels have emerged more than a billion years ago in an eukaryotic ancestor of choanoflagellates and metazoans [103].

In mammals, the functional diversity in T-type channels arises not only from the three genes expressing Cav3 isoforms with distinct electrophysiological properties [13, 28] but also from several alternative splicing events [56, 98, 99, 118]. Alternative splicing can generate multiple variants from a single Cav3 isoform with significantly distinct electrophysiological properties and drug sensitivity [25, 26, 54, 83, 101, 105, 132, 172]. Also, alternative splicing can regulate the Cav3 channel expression at the plasma membrane [133]. Alternative splicing could contribute to the clinical severity of Cav3 channelopathies, as documented by in vitro studies showing that disease-associated mutations exhibit distinct electrophysiological properties when reproduced in different splice variants [66, 122].

The tissue-specific expression of the Cav3 channels is clearly important to consider when investigating their physiological roles, as well as their implication in disease phenotypes [131]. In mammals, all Cav3 channels are expressed early during development. In adult, the three Cav3 isoforms are expressed mainly in the central and peripheral nervous systems and also in neuroendocrine and cardiac tissues [101, 102]. Within the brain, in situ hybridization studies have shown that the three Cav3 isoforms display both specific and distinct patterns of expression [12, 144]. In addition, Cav3 splice variants can be expressed in a tissue/cell-specific manner and be developmentally regulated [118]. Until now, the lack of highly specific antibodies for any of the Cav3 isoforms/variants has hampered precise analysis of their tissue and cellular and subcellular distribution at the protein level [1, 100, 166], which was partly circumvented by the generation of knock-in (KI) animals carrying epitope-tagged Cav3 channels [8, 58].

### Cav3 physiology

A hallmark of Cav3 channels is their unique ability to control neuronal excitability, requiring small membrane depolarizations to open (LVA), which distinguishes them from the high-voltage activated (HVA) channels [108, 168]. Their low threshold of voltage activation, coupled with their tonic inactivation near resting membrane potential, allows Cav3 channels to deinactivate and to underly the low-threshold spike/rebound bursting phenomenon seen in many types of neurons (Fig. 1a). The three Cav3 isoforms, which exhibit distinct electrophysiological properties [13, 28] (Fig. 1b), regulate differentially neuronal excitability [12, 39, 100]. In addition, the Ca^2+^ influx through Cav3 channels can also directly regulate intracellular Ca^2+^ concentrations [24, 51]. Indeed, all three Cav3 channels display an overlap of their steady-state inactivation and activation properties giving rise to a window current (Fig. 1c) that ressembles a “background” Ca^2+^ current [153]. It results from the activity of a small fraction of Cav3 channels remaining open in the voltage range near the resting membrane potential [34, 40]. The physiological role of this Cav3 window current is still poorly understood. It was shown to contribute to the slow oscillation in non-REM sleep [46].

Genetic manipulation of Cav3 expression in the mouse has provided significant information regarding the physiological roles of neuronal Cav3 channels and a quick overview of the findings obtained with Cav3 knock-out (KO) mouse models is provided here. In KO mice for *Cacna1g* (Cav3.1^−/−^), no LVA T-type current could be recorded in thalamocortical relay neurons and these neurons showed no burst firing activity [81] (Fig. 1a). In these animals, spike-and-wave discharges that occur in absence epilepsy models were prevented. The loss of thalamocortical oscillations was also observed in central medial nucleus, which reflects the overall importance of Cav3.1 channels in thalamic neurons [146]. Cav3.1^−/−^ mice were less prone to develop tonic seizures in the maximal electroshock seizure test, compared with wt littermates and Cav3.2^−/−^ mice, suggesting a prominent role of the Cav3.1 isoform in mediating tonic seizure [127]. Interestingly, overexpression of the Cav3.1 channel in a *Cacna1g* transgenic mouse line results in a pure absence epilepsy phenotype with no ataxia or other neurological disturbances [57], suggesting that an increase in Cav3.1 current is sufficient to the pathogenesis of spike-wave seizures. Cav3.1^−/−^ animals display a deficit in motor performance and in cerebellar learning [23, 94] and are resistant to harmaline-induced tremor [115]. In these animals, the T-type current was also significantly reduced in the subiculum, which is involved in hippocampal-dependent cognitive processes [79].

The KO mice for *Cacna1h* (Cav3.2^−/−^) exhibit a variety of phenotypes including neurological deficits [36]. The nociceptive role of Cav3.2 channels, which are highly expressed in the dorsal root sensory neurons [10, 11, 136], was validated using these Cav3.2 KO animals [41, 147] and further established when Cav3.2 channels were selectively deleted in low-threshold mechanoreceptor primary afferent neurons [58]. In the brain, Cav3.2 is predominantly expressed in the dentate gyrus of the hippocampus [1, 8]. In Cav3.2^−/−^ animals subjected to pilocarpine-induced status epilepticus, which models temporal lobe epilepsy, the appearance of epileptic seizures was strongly attenuated, validating the pro-epileptogenesis role of upregulated hippocampal Cav3.2 channels [7, 151]. In addition, Cav3.2^−/−^ mice show elevated anxiety and impaired hippocampus-dependent contextual memory and learning [38, 59].

Inactivation of *Cacna1i* in the mouse (Cav3.3^−/−^) resulted in the loss of LVA Ca^2+^ currents in the thalamic reticular nucleus (nRT) neurons and revealed a role of Cav3.3 channels in sleep. Cav3.3 channels dominate nRT rhythmogenesis and play a role in sleep spindles, the electroencephalographic hallmark of non-rapid eye movement (NREM) sleep [5, 85]. Of note, experiments performed in double Cav3.3 and Cav3.2 KO mice revealed that the lack of Cav3.2 channels further aggravates neuronal, synaptic, and EEG deficits in the Cav3.3^−/−^ background, indicating a role of Cav3.2 channels in regulating nRT excitability and rhythmogenesis [116, 145].

### Cav3 modulation

There is no evidence for a requirement of protein-protein association to obtain “native-like” properties of T-type channels when the Cav3 proteins are expressed in heterologous systems, as the HEK-293 cell line. This is contrasting with HVA Ca^2+^ channels, the L-type Cav1.1 to Cav1.4, and neuronal Cav2.1 P/Q-type, Cav2.2 N-type and Cav2.3 R-type, which require the auxiliary α2/δ, β, and γ subunits for their proper expression and function (reviewed in [108, 168]). However, a regulatory role of several proteins was identified for Cav3 channels, including the HVA auxiliary subunits [52], Kelch-like 1 [3], Stac1 [126], or the putative “Ca^2+^ channel and chemotaxis receptor domain containing 1,” CACHD1 [43] (Fig. 2). Regulation of Cav3 channels by such endogenous proteins would more likely reflect the numerous signalling pathways targeting Cav3 channels, as reported for the G protein βγ-dimer [50, 162], calmodulin [33, 86], syntaxin-1A [159], and spectrin α/β and ankyrin B [61] (Fig. 2).

The fine tuning of the functional properties of Cav3 channels by a large variety of endogenous pathways and ligands is now well established [29, 73, 75, 170] (Fig. 2). One of the first endogenous modulations described for Cav3 channels was the inhibitory effect of the endocannabinoid anandamide [27]. Other bioactive lipids, including arachidonic acid [143], N-acyl ethanolamides and polyunsaturated fatty acids [31], or 5,6-EET [21], were shown to also inhibit Cav3 channels in the micromolar range through a direct interaction [32]. Cav3 channels are also modulated by phosphorylation pathways, including the serine/threonine kinases, PKA and PKC [30, 71], Ca^2+^/CaM-dependent protein kinase II (CaMKII) [4, 161], rho-associated kinase (ROCK) [76], CDK5 [63], exchange factor activated by *cAMP* (*Epac*) [111], and hypoxia-inducible factor (HIF) [18]. As demonstrated for Cav3.2 channels, the phosphorylation status greatly influences the gating properties [9]. Phosphorylation also regulates an activity-dependent Ca^2+^ inhibition recently discovered for Cav3 channels, especially Cav3.3 [22, 35]. Other post-translational modifications regulating Cav3 channels, here Cav3.2 channels, including ubiquitination [60] and glycosylation [114, 160] have also been described. These latter studies point out that Cav3 isoform-specific modulations exist and are important to investigate further as they represent physiologically relevant selective regulations (Fig. 2). Of interest, the metal/redox modulation of T-type channels is also Cav3.2-specific. Cav3.2 channels are selectively upregulated by reducing agents such as l-cysteine, while the oxidizing agent ascorbate produces Cav3.2 channel inhibition [148]. This redox regulation occurs through the metal-catalyzed oxidation of a histidine residue (His191 in the human isoform) localized in the extracellular S3–S4 linker of domain I of Cav3.2 channel [72, 80]. Cav3.2 channels are also preferentially inhibited by the trace metal zinc (Zn^2+^) with an IC_50_ in the submicromolar range (∼ 0.8 μM), which is 100- and 200-fold lower than Cav3.1 and Cav3.3 channels, respectively [149]. Further studies, using a KI mouse model, have demonstrated that His191 is important for fine tuning of neuronal excitability in dorsal root sensory neurons [156].

## Cav3 channelopathies

### *CACNA1G*/Cav3.1 in late-onset cerebellar ataxia ADCA/SCA42

**Fig. 4:**
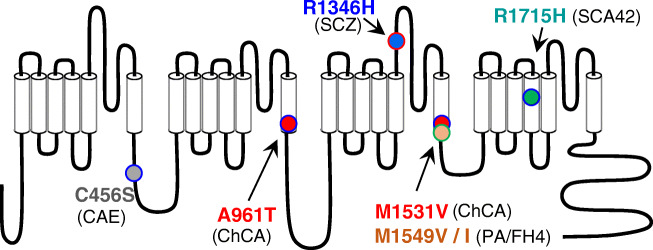
Schematic localization of the main Cav3 mutations described in the text, including (i) the Cav3.1 mutations: p.Arg1715His in SCA42 [44, 65], p.Arg961Thr and p.Met1531Val in ChCA [6, 34]; (ii) the Cav3.2 mutations: p.Arg1549Val and p.Arg1549Ile in PA/FH4 [47, 130], and p.Cys456Ser in CAE [53, 155]; and (iii) the Cav3.3 mutation: p.Arg1346His in SCZ [2, 62]

The Cav3.1 channel is highly expressed in the cerebellum, especially in Purkinje neurons [144], and was therefore a likely candidate for cerebellar disorders, especially ataxia. Hereditary cerebellar ataxias are rare neurodegenerative disorders, characterized by a cerebellar syndrome (gait alteration, limb incoordination, dysarthria, eye movement anomalies) with or without other neurological symptoms [45]. Using linkage analysis and whole-exome sequencing, *CACNA1G* was linked to an autosomal dominant cerebellar ataxia (ADCA) phenotype in three families, supporting its implication in spinocerebellar ataxia SCA42 [44] (Fig. 3). A recurrent missense mutation causing the p.Arg1715His substitution in the voltage sensor S4 segment of domain IV (IVS4, Fig. 4) of Cav3.1 was identified in these three unrelated pedigrees. This p.Arg1715His mutation affects the gating properties of the Cav3.1 channel with the steady-state activation properties shifted positively when expressed in HEK-293 cells. The expected reduction in channel activity was confirmed using computer modeling in deep cerebellar nuclei neurons that showed a decreased neuronal excitability. SCA42 is characterized by a slowly progressive ataxia with a variable onset but mainly in young adulthood. Athough the prevalence of SCA42 is very low, the association of this p.Arg1715His-Cav3.1 mutation with SCA42 was subsequently confirmed in Japanese and Chinese families [82, 88, 104, 109]. Additional *CACNA1G* missense mutations have been identified in other SCA42 patients, including p.Arg1068Cys, p.His1611Gln, and p.Pro2273His variants. However, they have shown no statistically significant electrophysiological effect in heterologous expression systems [44], while the variant p.Met1574Lys [88] has not been yet electrophysiologically explored. These additional *CACNA1G* variants clearly require further functional analysis to validate them as SCA42-causative mutations. Recently a KI mouse model of SCA42 was generated [65], harboring the above described mutation (p.Arg1723His in the mouse). Both heterozygous and homozygous KI mice demonstrated an adult-onset mild ataxia phenotype with comparable levels of motor impairment using rotarod and footprint tests, confirming the dominant inheritance of SCA42. Significant Purkinje neuron loss and degeneration of the molecular layer were also observed. This mouse model of SCA42 recapitulates well the observations made in SCA42 patients, as well as the electrophysiological analyses showing a positive shift of the voltage dependence of Cav3.1 channels [44]. Overall, this study confirms that SCA42 is caused by the p.Arg1715His mutation in Cav3.1 [65].

### *CACNA1G*/Cav3.1 in childhood cerebellar atrophy

Another set of de novo *CACNA1G* mutations was identified in a cohort of infants with childhood cerebellar atrophy (ChCA), using a combination of candidate gene panel and whole-exome sequencing [34]. ChCA is a devastating infantile neurodevelopmental disorder, with severe motor and cognitive impairments, cerebellar atrophy, and variable features including facial dysmorphism, digital anomalies, microcephaly, hirsutism, and epilepsy. Two mutations were identified in four individuals, three patients carrying a p.Ala961Thr mutation (in the Cav3.1 IIS6 segment) and one patient carrying a p.Met1531Val mutation (in the Cav3.1 IIIS6 segment) [34] (Fig. 4). Both mutations drastically altered the channel gating properties, especially the inactivation properties, with a significant slowing of the inactivation kinetics (5 times) and a negative shift (− 10 mV) of the potential for half-inactivation. In addition, these two mutations promoted a much larger window current that was fully inhibited by TTA-P2, a selective Cav3 channel blocker [34]. Overall, this study has demonstrated that p.Ala961Thr and p.Met1531Val are gain-of-function mutations. Importantly, this first description of de novo dominant *CACNA1G* mutations causing ChCA was confirmed by a recent study reporting on the same mutations (p.Ala961Thr and p.Met1531Val), identified in three patients and in one patient, respectively [6], strengthening the recurrence of these mutations in ChCA. The devastating consequence of ChCA gain-of-function mutations in humans reveals that Cav3.1 channel has a critical role in setting up cerebellar physiology during development. Further studies are necessary to uncover the pathogenic mechanism underlying the ChCA condition and to establish more precisely the developmental and functional roles of Cav3.1 channels in the cerebellum.

### *CACNA1G*/Cav3.1 in epilepsy

Because of their presence in cortical and thalamic structures and their role in modulating neuronal firing, T-type channels have always been considered candidates for idiopathic generalized epilepsies (IGEs). Interestingly, it was shown that *Cacna1g* is a genetic modifier of epilepsy in a mouse model of Dravet syndrome caused by mutations in the voltage-gated Na^+^ channel gene *Scn1a* [16], as well as a modifier in a *Scn2a* mouse model of focal epilepsy [15]. In humans, mutations in *CACNA1G* have been reported in juvenile myoclonic epilepsy patients [134]. However, the two reported missense mutations coding for p.Ala570Val and p.Ala1089Ser substitutions had no electrophysiological effect when explored in heterologous expression system, questioning their pathogenic status on a monogenic basis. A recent study has documented that IGEs have complex (oligogenic or multigenic) inheritance patterns with a likely combination of both common and rare genetic risk variants required to cause the disease. Among them, *CACNA1G*, carrying a high number of missense variants in IGEs samples, still represents a susceptibility gene [55].

### *CACNA1G*/Cav3.1 in other neurological diseases

Potential disease-causing variants in *CACNA1G* have also been identified in intellectual disability/cognitive disorders [106] and monoallelic deletions of the *CACNA1G* gene have been associated with mild intellectual disability without cerebellum atrophy [123]. Also, *CACNA1G* was identified as a candidate gene for autism spectrum disorder (ASD) in a subset of cases [142] but the *CACNA1G* association with ASD has yet to be replicated in a larger study [48]. *CACNA1G* also appeared to be a candidate gene in essential tremor, one of the most common movement disorders, with *CACNA1G* variants identified in three families [113]. It is therefore likely that the clinical spectrum of diseases associated with *CACNA1G* mutations will increase in a near future.

### *CACNA1H*/Cav3.2 in epilepsy

The *CACNA1H* gene, as *CACNA1G*, has received much attention regarding its potential implication in inherited epilepsy phenotypes. In some spontaneous mouse models of generalized epilepsy, the *tottering* (*tg*), *lethargic* (*lh*), and *stargazer* (*stg*) mouse strains, an increase in T-type current density was observed [169]. Strikingly, in the GAERS rat (*Genetic Absence Epilepsy Rats from Strasbourg*), a missense mutation, p.Arg1584Pro in *Cacna1h*, was found to co-segregate with the slow-wave discharge phenotype [150]. In heterologous expression system, this missense substitution in the intracellular loop linking the domains III to IV (LIII–IV) could induce a gain of channel activity when introduced in a Cav3.2 splice variant containing exon 25 [14, 122].

In humans, several studies have reported associations between *CACNA1H* single nucleotide polymorphisms (SNPs) and epileptic phenotypes, especially in idiopathic generalized epilepsy (IGE) [42, 69, 87, 138], reviewed in [158, 167], since the first report by Chen et al. [37] describing *CACNA1H* SNPs in childhood absence epilepsy (CAE) patients. Functional studies of several of these *CACNA1H* missense variants revealed that they could modify biophysical properties or protein trafficking of Cav3.2 in heterologous expression systems [69, 154, 155], in a loss- or gain-of-function manner. Many of these missense variants were found in the intracellular loop linking the domains I and II (LI–II) of Cav3.2 and one of them, p.Cys456Ser (Fig. 4), significantly increased spontaneous firing and reduced the threshold for rebound burst firing, when overexpressed in hippocampal neurons [53]. Yet, to date, none of these *CACNA1H* variants has been undoubtedly identified as causing seizure phenotypes. In other words, *CACNA1H* variants are not causing monogenic epilepsy [17]. As for *CACNA1G*, *CACNA1H* variants should be considered a risk factor for developing epilepsy, most likely implicating other genetic and/or environmental factors [67].

### *CACNA1H*/Cav3.2 in other neurological diseases

Four missense variants in the *CACNA1H* gene were identified in six individuals with ASD and the corresponding Cav3.2 variants showed altered electrophysiological properties in heterologous expression [140]. Whether these *CACNA1H* variants segregate with the ASD phenotype remains to be validated as these variations have low penetrance and some of them were also found in unaffected individuals. Additional *CACNA1H* variants were reported in a patient with persistent pain [137] and in patients with amyotrophy lateral sclerosis [125, 141]. Again, further studies are needed to validate association of *CACNA1H* variation with these conditions.

### *CACNA1H*/Cav3.2 in primary aldosteronism

Using whole-exome sequencing, Scholl et al. [130] identified a recurrent missense mutation, p.Met1549Val, in the *CACNA1H* gene in five unrelated patients from a cohort of patients diagnosed with primary aldosteronism (PA) in early childhood (Fig. 3). This point mutation resulted in a significant gain of Cav3.2 channel activity. Soon after, Daniil et al. [47] also performing whole-exome sequencing in PA patients reported another substitution at this residue, p.Met1549Val, with similar gain-of-function properties. Additional gain-of-function mutations, p.Ser196Leu, p.Pro2083Leu, and p.Val1951Glu, were also identified in this study [47]. If one patient was diagnosed with minor mental retardation and multiplex developmental disorder, other patients showed no apparent signs of seizures, cardiac arrhythmia, or muscular or neurological alterations. *CACNA1H*-related PA is now defined as familial hyperaldosteronism type 4 (FH4; [120]).

The Met1549 amino acid is located in highly conserved sequence of the IIIS6 segment of Cav3.2 that lines the inner part of the channel pore and is involved in channel inactivation [97] (Fig. 4), indeed at the same position as Met1531 in Cav3.1 (Fig. 3). The two pathogenic substitutions of Met1549 (Val and Ile) confer ultraslow inactivation kinetics, significant negative shift in the steady-state inactivation properties, and an increased window current. These data support an increase in channel activity and a rise in intracellular Ca^2+^ [47, 130]. Notably, all these mutants led to increased aldosterone production and increased expression of the genes coding for steroidogenic enzymes in the adrenocortical H295R cell line after K^+^ stimulation [47, 124]. Additional *CACNA1H* mutations causing PA/FH4 will likely be identified, as exemplified by the recent description of a p.Ile1430Thr substitution (IIIS5 segment), in an aldosterone-producing adenoma [107].

### *CACNA1I*/Cav3.3 in neurological/psychiatric diseases

Genome-wide association studies (GWAS), as well as the identification of de novo variants in the *CACNA1I* gene, have contributed to implicate *CACNA1I* as a genetic risk factor in schizophrenia (SCZ) [64, 77, 129]. When expressed in the HEK-293 cell line, one of the two Cav3.3 missense variations identified in [64], p.Arg1346His (Fig. 4), resulted in a lower expression level of the Cav3.3 protein, a reduced N-glycosylation, and a reduced expression at the plasma membrane, reducing the Cav3.3 current but with no change in the electrophysiological properties [2]. A KI mouse model was generated using the CRISPR/Cas9 editing approach to introduce the p.Arg1305His orthologous mutation [62]. The homozygous animals show altered excitability in the nRT and deficits in sleep spindle occurrence and at NREM/REM transitions. This animal model will facilitate further investigations of the role of Cav3.3 channels in impaired sleep spindle and nRT function in SCZ. Additional *CACNA1I* variants have been identified in SCZ patients, confirming *CACNA1I* as a genetic risk factor in SCZ [95, 163, 164]. *CACNA1I* is also considered a risk gene in autism [93] and other complex neuropsychiatric disorders [128].

## Conclusions and perspectives

### De novo gain-of-function mutations in Cav3 channels: a wider group of S6-pathies?

These last years, many novel disease-related Cav3 channel variants have been reported and some of them are causing severe disorders. This is the case for the de novo gain-of-function mutations in Cav3.1 and Cav3.2 channels in childhood cerebellar atrophy (ChCA) and primary aldosteronism (PA/FH4), respectively. These deleterious missense mutations involve residues, Ala961 and Met1531 in Cav3.1 and Met1549 in Cav3.2, in the highly conserved S6 segments lining the inner part of the pore channel (Fig. 4). These residues were implicated in Cav3 channel inactivation in earlier structure-function studies [49, 97]. Notably, these “S6 mutations” in Cav3.1 and Cav3.2 are reminiscent to several de novo gain-of-function mutations recently described in other genes encoding Cav channels (Fig. 3). These other Cav “S6 mutations” also cause severe, mainly neurodevelopmental, clinical phenotypes. S6 mutations in *CACNA1C*, which encodes the Cav1.2 L-type channel, cause Timothy syndrome (TS), a congenital long-QT cardiac arrhythmia with or without severe neurological phenotypes, including autism and mental retardation [89, 139]. There are S6 mutations in *CACNA1D*, which encodes Cav1.3, another L-type channel, that causes a neurodevelopmental disorder including ASD, intellectual disability with or without neurological (hypotonia, epilepsy) and endocrine (primary aldosteronism or hyperinsulinemic hypoglycaemia) features (PASNA) [70, 121]. There are also S6 mutations in Cav2.3, the neuronal R-type channel encoded by *CACNA1E*, that causes developmental and epileptic encephalopathies (DEE) [68]. There is also recent evidence for de novo S6 mutations in *CACNA1A*, encoding the neuronal P/Q-type Cav2.1 channel, linked to severe DEE with intellectual disability and variable motor symptoms [78]. All these S6 missense mutations share functional features: they significantly impair the inactivation properties of the affected Cav channels, likely promoting increase in intracellular Ca^2+^ concentration and the subsequent cellular damages caused by abnormal Ca^2+^ homeostasis [96]. Considering the similarity in their pathogenic mechanism, we tentatively propose here to define this group of Ca^2+^ channelopathies as “S6-pathies.” Further studies, exploiting animal models of the corresponding channelopathies, will help to identify the pathogenic mechanisms underlying the diseases and better delineate the precise implication(s) of the corresponding Cav channels. Deciphering the role of Cav3.1 and Cav3.2 in ChCA and PA/FH4, respectively, should benefit from a combined effort of the “calcium channelopathy” community.

### Structural studies of Cav3 channels: further deciphering of the disease mechanisms

High-resolution structural studies can provide atomic-level views of disease mechanisms [20]. Notably, the Cryo-EM structure of the Cav3.1 channel was recently reported [171], opening new opportunities to better understand the molecular and functional consequences of disease mutations in Cav3.1 channel, as well as in Cav3.2 and Cav3.3 channels by homology modeling. It is also anticipated that the pharmacology of Cav3 channels will benefit from the development of novel therapeutic approaches using structure-guided drug discovery. Cav3/T-type channels have always been considered promising pharmacological targets considering their implication in a wide variety of neurological conditions, including epilepsy and pain. However, until now, the clinical development of drugs targeting Cav3 channels has not been as successful as expected [92, 157], likely because of the wide tissue expression of the Cav3 channels, the lack of selective Cav3 channel blockers, and, beyond that, the lack of Cav3 isoform-specific blockers. Further studies should establish whether there is a therapeutic potential of Cav3 blockers in the treatment of ChCA and PA/FH4 diseases that are directly caused by increased activity of the Cav3.1 and Cav3.2 channels.
